# The Association between the Distribution of Resin Beads and the Emergence of *Sirex noctilio* on Red Pine in North America

**DOI:** 10.3390/insects13121111

**Published:** 2022-11-30

**Authors:** Hajar Faal, Stephen A. Teale

**Affiliations:** 1 Department of Environmental and Forest Biology, College of Environmental Science and Forestry, State University of New York, Syracuse, NY 13210, USA; 2 Forest Pest Methods Laboratory, USDA-APHIS-PPQ, 1398 W. Truck Rd., Buzzards Bay, Barnstable, MA 02542, USA

**Keywords:** red pine, resin beads, cardinal direction, wood borers, *Sirex*, *Ibalia*

## Abstract

**Simple Summary:**

The European woodwasp, *Sirex noctilio*, is an invasive pest in North America. The presence of resin beads as signs of female ovipositor activity has been effectively used in ground surveillance. Improving our understanding of the relationship between resin bead density and the number of emerging *S. noctilio*, its parasitoid, and native woodwasp strengthens integrated pest management programs. We also investigated the resin bead density associated with the height, diameter, or cardinal direction on red pines. Results indicated resin bead density was significantly associated with only the emergence of *S. noctilio*, not the emergence of its parasitoid, or native woodwasp. Resin beads were also most abundant on the north, east, and south sides of logs at 4.5 m above the ground. This study supports the use of resin beads as a first visible sign of *S. noctilio* colonization of host trees in North America.

**Abstract:**

This study examined the relationships of the abundance and distribution of resin beads (signs of *Sirex noctilio* parent female ovipositor activity) with the abundance and distribution of emerging progeny of *S. noctilio*, *S. nigricornis* and their parasitoid *Ibalia leucospoides*. *S. noctilio* is native to Europe and is an invasive pest of pines in the Southern Hemisphere and North America; *S. nigricornis* is native to North America and is a secondary pest of dying pines. *I. leucospoides* is a parasitoid that has been widely deployed for biological control of *S. noctilio*. This study aimed to determine if the distribution of resin beads is associated with the height, diameter, or cardinal direction on red pines, *Pinus resinosa*, as well as the distribution of wood wasp and parasitoid emergence. Our results showed that among log sections taken at five heights, resin beads were most abundant on the north, east, and south sides of logs and mid log at 4.5 m above the ground. Emergence of *S. noctilio* was most abundant only from logs with more than five resin beads per square meter, while diameter and height were not contributing factor. None of variables evaluated (resin bead densities, height, and diameter) had significant effects on the emergence of *S. nigricornis* and *I. leucospoides*. These findings help clarify the biological significance of resin beads as indicators of *S. noctilio* colonization of host trees in North America.

## 1. Introduction

The Eurasian woodwasp, *Sirex noctilio* (Hym.: Siricidae), is an invasive wood borer that is attracted to physiologically stressed pine trees of suppressed and intermediate crown position [1]. Physiological stresses intensify tissue permeability and result in enhanced monoterpene production [2], which increases the probability of *S. noctilio* attack. Monoterpene composition influences host selection by *S. noctilio*, and the amount and duration of monoterpene production are affected by tree resistance as well as the degree and duration of stress [3,4].

*S. noctilio* attack induces increased biosynthesis and accumulation of resin [5]. The resin fills the *S. noctilio* drill holes and egg galleries while a small amount flows to the bark surface forming a droplet which crystallizes into a resin bead [6,7,8]. However, resin pressure declines in stressed hosts [2], and *S. noctilio* attack intensifies the original stress and causes an additional drop in resin flow by injecting a phytotoxic venom (mucus) and a pathogenic fungus, *Amylostereum areolatum*, in their drill holes [9]. Due to the toxicity and quantity of venom, as well as the pathogenicity of the fungus, *S. noctilio* is the only siricid with the ability to overcome tree defenses and eventually kill the host tree [10].

Native *Sirex* species, wood boring beetles, and bark beetles commonly occur in the same host with *S. noctilio* [11,12,13,14,15,16,17,18,19,20]. *S. nigricornis*, a native siricid in North America, typically attacks dying pine trees [21], but may occur in trees attacked by *S. noctilio* because it exploits the fungal symbiont deposited by *S. noctilio* [20]. Subcortical interspecific competition can adversely influence biological control programs using the nematode *Deladenus siridicola*. For instance, *Ips* bark beetles often arrive first and promptly inoculate trees with blue stain fungi [15,16,22]. In addition, wood-boring beetle larvae directly compete with *S. noctilio* larvae for nutrition and space [23] or feed on *S. noctilio* larvae [24]. These arboreal pests may limit the access of *S. noctilio* to nutrition sources, depending on which species arrives first [23].

The parasitic wasp, *Ibalia leucospoides* (Hymenoptera: Ibaliidae), has been widley introduced as a biological control agent against *S. noctilio* in the Southern Hemisphere [25,26]. The extensive use of *Ibalia* as a parasitoid in *Sirex* management prompted many investigations into its biology and ecology [26,27,28]. *Ibalia* females use the oviposition holes of siricid woodwasps to access their host [1,29,30], but it is not known if, or to what extent, resin beads interfere with the parasitoid’s access to late-stage eggs and early instar larval siricids.

Resin beads, as the first visible sign of *S. noctilio* attacks, are the most effective visual cue used in ground surveillance to locate infested host trees. The present study aims to improve our understanding of the relationship between resin bead density and distribution and the number of emerging *S. noctilio*. The objectives are to (i) examine whether the density of resin beads is associated with the cardinal directions, tree height, or tree diameter at different heights; (ii) compare the distribution of wood-boring wasps (*S. noctilio, S. nigricornis*, and *I. leucospoides*) emerging from trees with tree height, tree diameter, and the density of resin beads; and (iii) determine whether the emergence of siricids is influenced by the abundance of wood boring beetle galleries.

## 2. Materials and Methods

### 2.1. Distribution of Resin Beads

Twelve red pines, *Pinus resinosa*, with signs of *S. noctilio* infestation, were selected at Chimney Bluffs State Park (43°16′50″ N, 76°54′48″ W), 10 km nw of Wolcott, NY, USA in December 2017. Their crown class was observed and recorded, their height was measured using a clinometer and meter tape [31], and tree diameter was measured at breast height (DBH). Each tree was sampled at five 0.5 m long sections with midpoints at heights of 1.5, 3, 4.5, 6, and 7.5 m above ground. The circumference of each section was measured at the midpoint, and the density of resin beads was calculated on each section and recorded relative to cardinal direction. The density of resin beads in each cardinal direction quadrant were counted for each section. The sample sections were marked for subsequent dissection.

### 2.2. Distribution of Insects

On 26 March 2018, ten of the trees were felled and the marked sample sections were excised. The unsampled tree sections were discarded. Two trees were abandoned due to woodpecker damage. The 50 sampled sections were sealed with hot wax (Gulf Wax, Royal Oak Enterprises, LLC, Roswell, GA, USA). The log sections were individually placed in mesh bags and kept at room temperature. Beginning with the first emerging wasp, emergence was recorded daily for six weeks, and subsequently once weekly until the end of September 2018. Then, each bolt was split to a maximum thickness of 1 cm using an electric log splitter (Grizzly Industrial, Inc., Model H8170/H8171, China). All live and dead wood borer larvae and adults were recorded as well as the presence or absence of blue stain fungi. The number of unemerged wasps for each section was estimated using methods of Standley (2012). The galleries of wood boring beetle larvae were extensively coalesced and difficult to count, so only the presence or absence of galleries was recorded for wood boring beetles.

### 2.3. Data Analysis

#### 2.3.1. Distribution of Resin Beads

The density of resin beads was calculated for each sampled section and cardinal direction. The trunk diameter (X) was categorized into three groups, 10–15, 15–20, or 20–25 cm in diameter. The distribution of resin beads as a function of tree height and diameter, and cardinal direction was evaluated using Generalized Linear Models (GLM) with a negative binomial distribution and the least squares means (LSMeans) statement in SAS 9.4.

#### 2.3.2. Distribution of Insects

The distribution of *S. noctilio*, *S. nigricornis*, *I. leucospoides* emergences was evaluated as a function of height, trunk diameter, and resin bead density (fixed effects), while individual trees were treated as a random effect. Resin bead densities (Y) were categorized into four groups: 0, 0–5, 5–10, >10 resin beads per square meter. The effect of resin bead density on wood borer survival was first assessed using Generalized Linear Mixed Models (GLMM) with a Poisson distribution and logarithmic link function, but the Poisson regression was replaced with a negative binomial due to overdispersion and formal Akaike information criterion (AIC) model selection. The model coefficient (β) indicates a change in the independent variable. The expected response variable changes by a multiplicative factor of exp(β) for each unit change of an independent variable if the other independent variables in the model are held constant. GLM with a negative binomial distribution was applied to the significant response variables derived from GLMM to distinguish which level of the independent variable had a significant effect on the response variable. Degrees of freedom for the fixed effects F-test were adjusted for statistical dependence using Satterthwaite formulas.

## 3. Results

### 3.1. Distribution of Resin Beads

**Figure 1:**
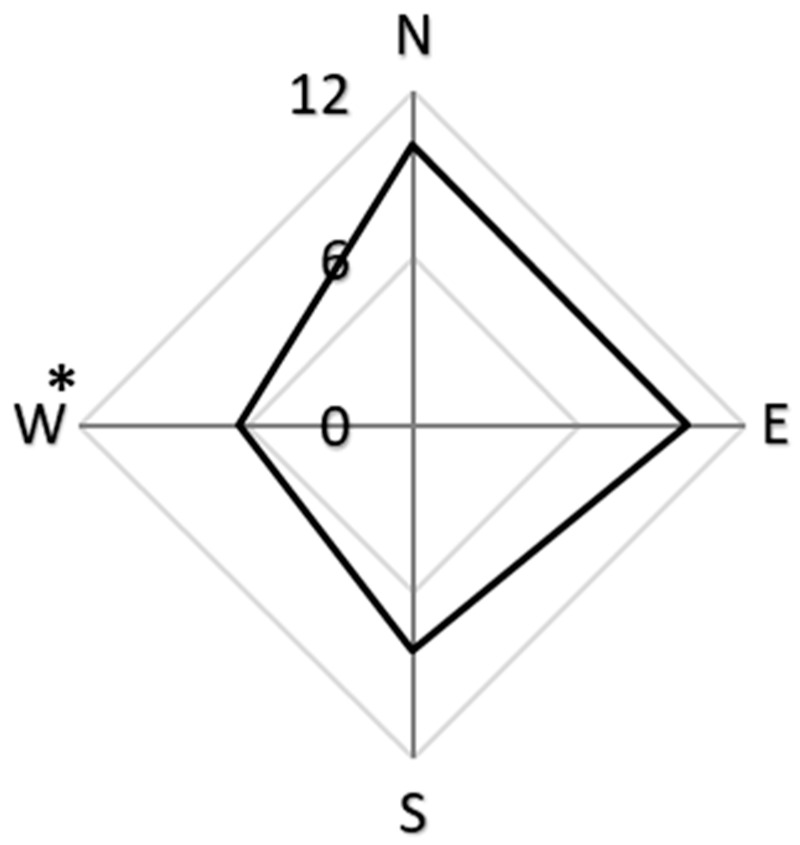
The density of resin beads per square meter on red pines, *Pinus resinosa*, by cardinal direction. The asterisk (*) indicates significantly lower resin bead density (*p* = 0.04).

**Figure 2:**
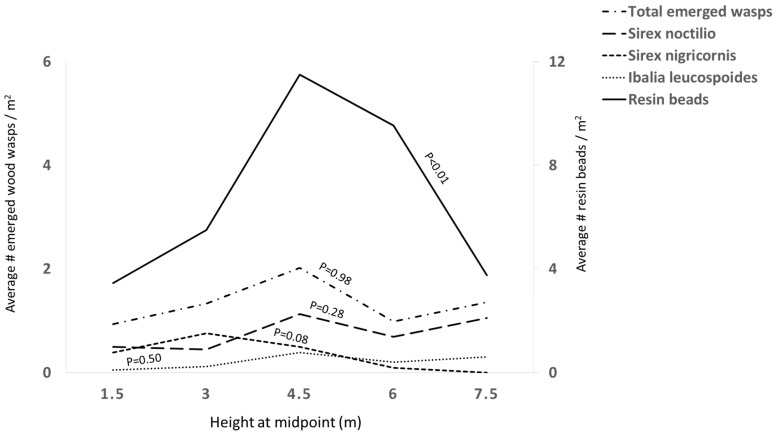
The density of resin beads and emerged wood wasps per square meter on log sections with heights of 1.5, 3, 4.5, 6, and 7.5 m above ground. The height was significant only for resin bead density (*X*^2^ = 21.63, *p* < 0.01), not for the emergence of *Sirex noctilio* (*p* = 0.28), *S. nigricornis* (*p* = 0.08), and *Ibalia leucospoides* (*p* = 0.50).

**Figure 3:**
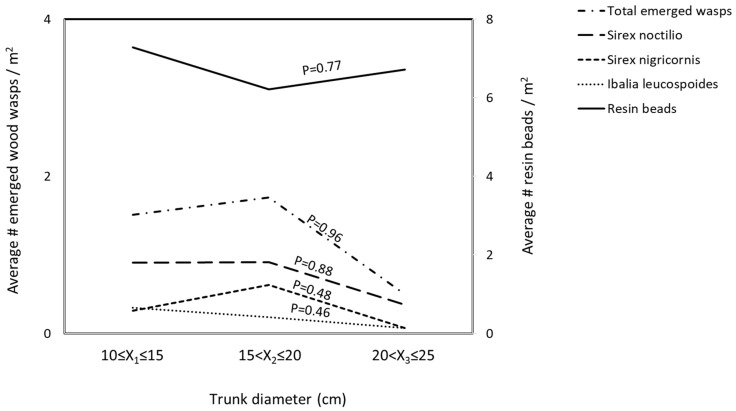
The density of resin beads and the average number of emerged wood wasps per square meter on log sections of red pines, *Pinus resinosa*, by diameter (X). Log diameter was not a significant factor influencing resin bead density (*p* = 0.77), nor the emergence of *Sirex noctilio* (*p* = 0.88), *S. nigricornis* (*p* = 0.48), *Ibalia leucospoides* (*p* = 0.46), and the total wasps (*p* = 0.96).

The sampled red pines had suppressed and intermediate crowns, heights of 9–12 m and DBH of 15.24–28 cm. The density of resin beads ranged from 0 to 23 per square meter of log surface (average = 6.75 ± 0.82/m^2^). The density of resin beads was influenced by the cardinal direction (*p* = 0.04, *X*^2^ = 4.09) (Figure 1), the tree height (*p* < 0.01, *X*^2^ = 21.63) (Figure 2), but not trunk diameter (*p* = 0.77, *X*^2^ = 0.09) (Figure 3).

The majority of resin beads was observed on the north (10.1 resin beads/m^2^), east (9.9 resin beads/m^2^), and south (8.1 resin beads/m^2^), but not west (6.3 resin beads/m^2^) aspects of the log sections (Figure 1) and on the mid bole sections at 4.5 m (11.5 resin beads/m^2^), followed by 6 m above the ground (9.5 resin beads/m^2^) (Figure 2).

### 3.2. Distribution of Insects

*S. noctilio* and its parasitoid *I. leucospoides* simultaneously started to emerge on 18 May 2018 and continued emerging for four weeks, while emergence of *S. nigricornis* began three months later on 10 August and continued for two weeks. From fifty half-meter-long log sections with a cumulative surface area of 135.5 m^2^, the total numbers of emerged *S. noctilio*, *S. nigricornis*, and *I. leucospoides* were 101 (average = 0.82 ± 0.2/m^2^), 46 (average = 0.35 ± 0.1/m^2^), and 26 (average = 0.22 ± 0.1/m^2^), respectively. The average sex ratios (male to total) of *S. noctilio*, *S. nigricornis*, and *I. leucospoides* were 0.71 ± 0.07, 0.40 ± 0.12, and 0.62 ± 0.11, respectively.

**Figure 4:**
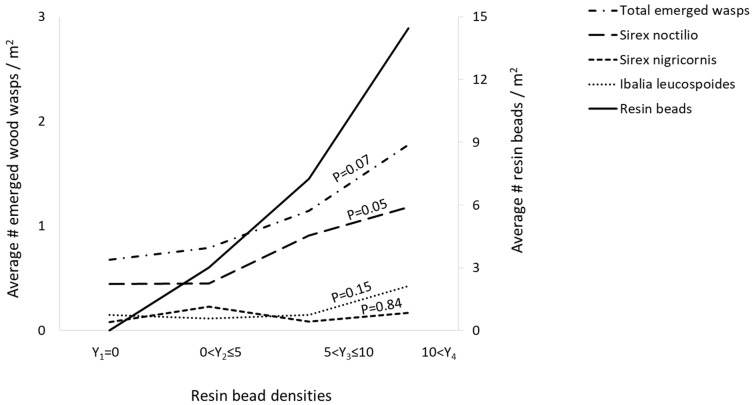
The mean number of emerged wood wasps per square meter collected from infested red pines, *Pinus resinosa*, versus the densities of resin beads (Y). The density of resin beads was significant only for the emergence of *Sirex noctilio* (*p* = 0.05), but not for *S. nigricornis* (*p* = 0.84), *Ibalia leucospoides* (*p* = 0.15), and total wasps (*p* = 0.07). A significant number of *S. noctilio* emerged from sections with higher resin bead densities.

The height of the log sections was not a significant variable for emerged *S. noctilio*, *S. nigricornis*, and *I. leucospoides* (Table 1, Figure 2). The log diameter did not affect the emergence of the three species (Table 1, Figure 3). A significant number of *S. noctilio* emerged from logs with more than five resin beads per square meter (*F* = 5.30, *p* < 0.01), however, the emergence of *S. nigricornis* and *I. leucospoides* was not affected by the density of resin beads (Table 1, Figure 4). None of the independent variables influced the total number of unemerged wasps (Table 1). There were no unemerged wasps in the log sections that were free of blue stain fungus and beetles.

Wood borer beetles preferred thicker parts of the trunk and there were no wood borer galleries in sections from the 7.5 m height. Wood borer beetles shared 42% of the log space with siricids, solely utilized 18% of the log sections, while *S. noctilio* colonized 24%. The blue stain fungi colonized the entire bolt in 9% of the total examined log sections and partially colonized the remaining log sections.

## 4. Discussion

The distribution of resin beads was influenced by tree height and cardinal direction. The density of resin beads was higher on the north, east, and south sides and on mid bole log sections 4.5 m above the ground. The density of resin beads was the only variable significantly associated with the number of emerged *S. noctilio*.

The density of resin beads varies between dominant and suppressed trees, and is related to several factors, including tree resistance to *S. noctilio* attacks [6,32], tree physiology [2,33], tree health, and prevailing winds [34,35,36,37,38].

Resinosis is the initial and fundamental barrier for *S. noctilio* to overcome, as evidenced by its high mortality in early stages of development [17]. The degree of tree resistance to *S. noctilio* attack can determine the distribution of resin beads; for example, Madden [8] reported random patterns on susceptible trees and aggregated patterns on resistant trees. We did not observe any aggregated patterns on the trees studied, and we did not evaluate tree resistance. Additionally, host susceptibility is related to tree age or size at the time of the attack; for instance, *S. noctilio* females prefer smaller diameter trees [39]. Thompson et al. [24]) also showed that *S. noctilio* made fewer oviposition wounds in larger trees than smaller trees. In the current study, tree diameter was not a significant factor for the emergence of any wasps.

Phloem and cambium physiology influence the number of *S. noctilio* drills and eggs [33]. In *P. radiata,* the rate of respiration varies with tree size and at different heights within a tree [2]. The maximum respiration rate occurs in trees with a diameter of 15 cm and between 2.5 to 4 m above the ground. The phloem in this area of the stem exhibits minimal diurnal fluctuations and maximal respiration. Any physiological disturbance first occurs in that vulnerable area, causing a change in tissue permeability and release of volatile monoterpenes that attract *S. noctilio* [2]. In our study, more resin beads were observed at heights of 4.5 m, which is a range similar to that described by Ryan et al. [40].

In the current study, more *S. noctilio* emergence from red pine logs with more resin beads may relate to tree health. Red pine can be a prime host for *S. noctilio* in the absence of Scots pine in the northeastern U.S. [41,42]. Red pine, *P. resinosa*, has been extensively planted in the northern U.S. and eastern Canada because of its ability to grow on poor quality sites [43]. Variation in red pine growth rates is strongly influenced by soil characteristics due to relatively low genetic diversity [44,45]. Red pines grow poorly or die in highly compacted, water-logged, or unfavorable pH soils. Chimney Bluffs State Park along to Lake Ontario, Wolcott, NY, USA was a prime location for this research because the *S. noctilio* population has been established in this location since at least 2010 [46]. This stand is unmanaged, and in 2011 the stand density and basal area were 1200 ± 113 tree/ha and 0.4 ± 0.03 m^2^/ha, respectively [46]. This suggests that the effect of poor-quality site may be a contributing factor in maintaining the population of *S. noctilio* in the northeastern U.S.

Insect activity is often more prevalent on the south facing side of host trees, where they exploit the extended exposure to sun in winter [47,48], or avoid bird predators [49] or parasitoids [50]. It is likely that wind drift and prevailing winds also influence insect activity, for instance bark beetles fly upwind towards pheromone sources located on the tree bole [34,35,36,37,38]. In this study, the minimum resin bead densities occurred on the west side of the trees studied, which may be related to the upwind orientation of insects toward semiochemical sources prior to landing [35]. Coutts [32] reported that *S. noctilio* mostly attacked the northeast side of host trees which is opposite the prevailing wind direction at that location. Thus, the orientation of insects to semiochemical sources may explain the radial distribution of resin beads.

The majority of subcortical insects spatially partition their habitats to maximize the exploitation of resources [51], for example bark beetles indirectly partition the tree by employing their symbiont, blue stain fungi, to colonize larger areas because they outcompete *A. areolatum* [15]. The failure of *A. areolatum* growth would directly obstruct the development of woodwasp larvae [52]. The blue stain fungi colonized the entire bolt in 9% of the total examined log sections and partially colonized the remaining log sections, and eventually impeded the development of other subcortical insects, either entirely or partially. This result is in agreement with Foelker et al. [53], who reported that a 10% increase in blue stain volume caused an 11.2% decrease in *S. noctilio* survival. Therefore, the competition between wood borers and bark beetles jeopardizes the survival and development of *S. noctilio*.

*S. noctilio* has a wide range of host plants with different resin canal densities [54]. Thus, describing factors that contribute to the success or failure of commericial hosts to produce resin beads in response to *S. noctilio* attacks may be fruitful topic to investigate. In the current study, we showed that the density of resin beads is a reliable indicator of the presence of *S. noctilio* red pines, *P. resinosa*, growing on a poor-quality site. By enhancing our understanding of the relationship between resin beads and the presence of *S. noctilio,* our results should improve surveillance of this important pest.

## Figures and Tables

**Table 1:** Results of Generalized Linear Mixed Model analysis for the wood borer wasps.

Variables	Emerged			Total Emerged Wasps	Total Unemerged Wasps
	*Sirex noctilio*	*Sirex nigricornis*	*Ibalia leucospoides*		
Resin beads					
β ^1^	0.48	−0.07	0.48	0.35	0.19
F	4.05	0.04	2.21	3.41	0.20
P	0.05 *	0.84	0.15	0.07	0.66
Df ^2^	1, 37	1, 37	1, 37	1, 37	1, 37
Height					
β	0.16	−0.40	0.14	0.02	−0.07
F	1.19	3.34	0.46	0.00	0.06
P	0.28	0.08	0.50	0.98	0.81
Df	1, 37	1, 37	1, 37	1, 37	1, 37
Diameter					
β	0.08	−0.53	−0.38	0.03	−0.37
F	0.02	0.49	0.54	0.00	0.30
P	0.88	0.48	0.46	0.96	0.59
Df	1, 37	1, 37	1, 37	1, 37	1, 37

^1^ β = Negative binomial regression coefficients. The sign of the coefficient represents the direction of effect of that independent variable on the response variable. ^2^ Degrees of freedoms (Num Df and Den Df) are indicated, respectively. Asterisks denote significant variables: * α = 0.05.

## Data Availability

The data presented in this study are openly available in Figure 1, Figure 2, Figure 3 and Figure 4, and Table 1.
